# Supplementary material for: Phenotypic pliancy and the breakdown of epigenetic polycomb mechanisms
Source: PLoS Comput Biol. 2023 Feb 21;19(2):e1010889. doi: 10.1371/journal.pcbi.1010889 (PMC9983867; doi:10.1371/journal.pcbi.1010889)
Supplement: S1 Fig — The average combined fitness for both environment 1 and environment 2 shown in the insert due to scale (green), and the average fitness for environment 1 (red) and environment 2 (blue) fitness results. (PDF) [file pcbi.1010889.s001.pdf]

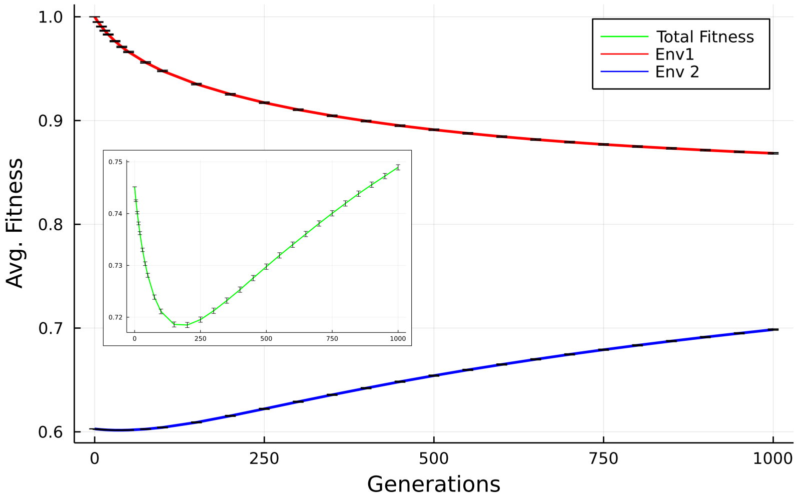

Fig S 1. Average Fitness Throughout Evolution: The average combined fitness for both environment 1 and environment 2 shown in the insert due to scale (green), and the average fitness for environment 1 (red) and environment 2 (blue) fitness results.
