# Supplementary material for: Phenotypic pliancy and the breakdown of epigenetic polycomb mechanisms
Source: PLoS Comput Biol. 2023 Feb 21;19(2):e1010889. doi: 10.1371/journal.pcbi.1010889 (PMC9983867; doi:10.1371/journal.pcbi.1010889)
Supplement: S2 Fig — The average number of genes that are repressed by any PRC during evolution in environment 1 (red) or environment 2 (blue). (PDF) [file pcbi.1010889.s002.pdf]

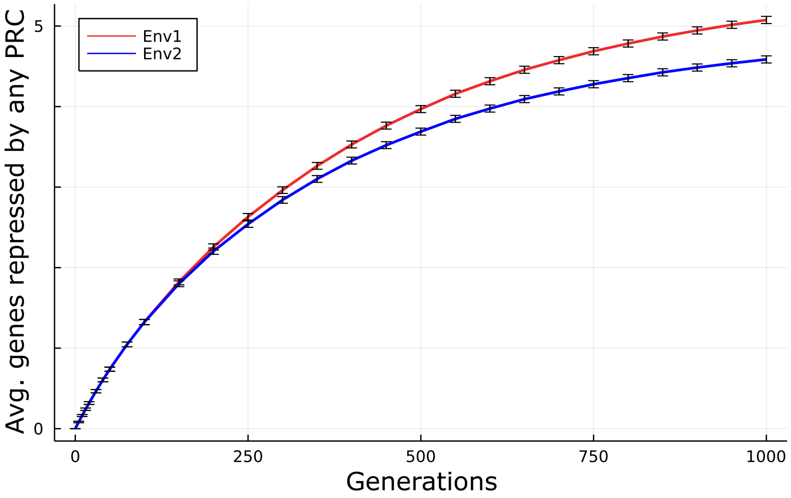

Fig S 2. Genes Repressed by PcG-like mechanisms During Evolution: The average number of genes that are repressed by any PRC during evolution in environment 1 (red) or environment 2 (blue).
