# Supplementary material for: Phenotypic pliancy and the breakdown of epigenetic polycomb mechanisms
Source: PLoS Comput Biol. 2023 Feb 21;19(2):e1010889. doi: 10.1371/journal.pcbi.1010889 (PMC9983867; doi:10.1371/journal.pcbi.1010889)
Supplement: S3 Fig — The average incoming (A) and outgoing (B) effective connectivity of the gene regulatory networks for all 10 million cells (solid lines). The effective connectivity averaged across all the genes in the network for environment 1 and environment 2 decreases throughout evolution as would expect (magenta and cyan solid lines). The average connectivity of the target PRC genes, whether repressed or not during development (green dotted lines), and only the genes repressed by any PRC (red and blue dotted lines) are shown as well. Genes that are repressed by any PRC have a higher average incoming connectivity as compared to all the target genes. Note, we look at average connectivity, as opposed to average effective connectivity, for PcG-like mechanism target genes that are repressed or not to show the connections the repressed target had before repression. (PDF) [file pcbi.1010889.s003.pdf]

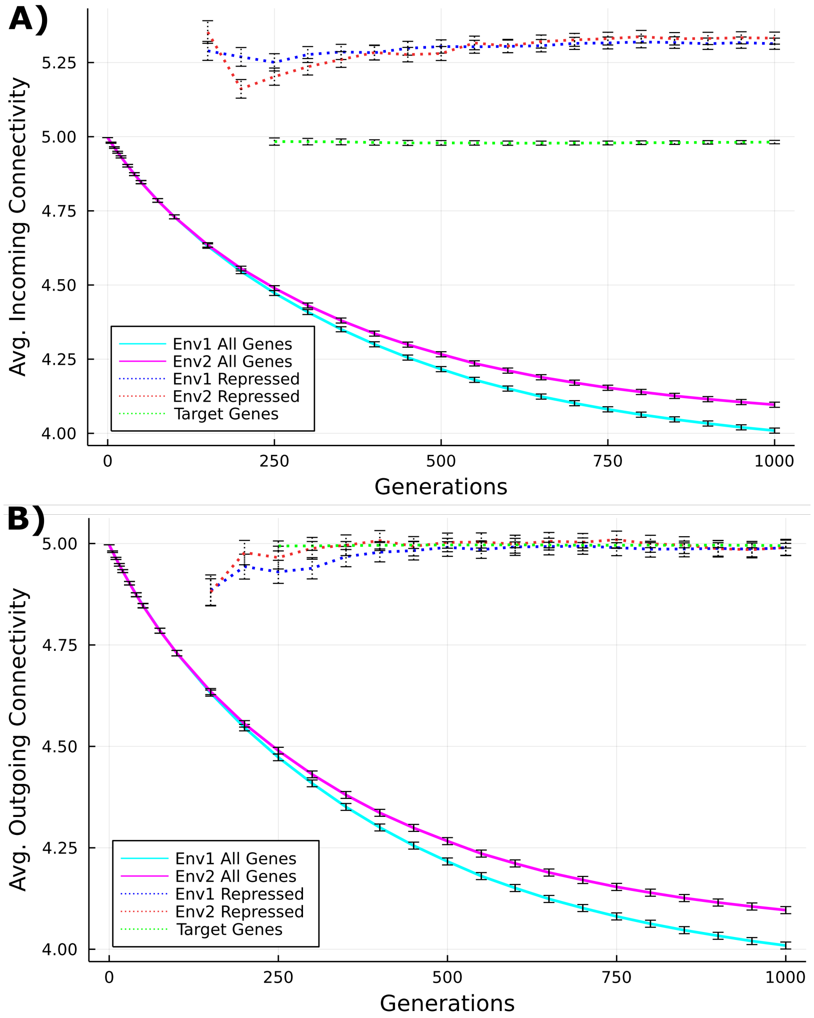

Fig S 3. Connectivity of the Gene Regulatory Networks Throughout Evolution: The average incoming (A) and outgoing (B) effective connectivity of the gene regulatory networks for all 10 million cells (solid lines). The effective connectivity averaged across all the genes in the network for environment 1 and environment 2 decreases throughout evolution as would expect (magenta and cyan solid lines). The average connectivity of the target PRC genes, whether repressed or not during development (green dotted lines), and only the genes repressed by any PRC (red and blue dotted lines) are shown as well. Genes that are repressed by any PRC have a higher average incoming connectivity as compared to all the target genes. Note, we look at average connectivity, as opposed to average effective connectivity, for PcG-like mechanism target genes that are repressed or not to show the connections the repressed target had before repression.
