# Supplementary material for: Phenotypic pliancy and the breakdown of epigenetic polycomb mechanisms
Source: PLoS Comput Biol. 2023 Feb 21;19(2):e1010889. doi: 10.1371/journal.pcbi.1010889 (PMC9983867; doi:10.1371/journal.pcbi.1010889)
Supplement: S4 Fig — PCA results for all individuals in population at the end of evolution (after generation 1,000) when evolve with no PcG mechanism evolution to assess phenotypic fidelity. After evolution when transfer from environment 1 to environment 2 (cyan) and compare to environment 1 (orange) and environment 2 (purple X’s), we see that the individuals move from environment 1 to environment 2 and thus do not exhibit phenotypic fidelity like they do when evolve with polycomb mechanism. Note, when evolve without polycomb and move from environment 1 to environment 2 (cyan), the results exactly overlap with environment 2 (purple X’s) because the networks for these cases are the same when do not have any polycomb repressed genes. (PDF) [file pcbi.1010889.s004.pdf]

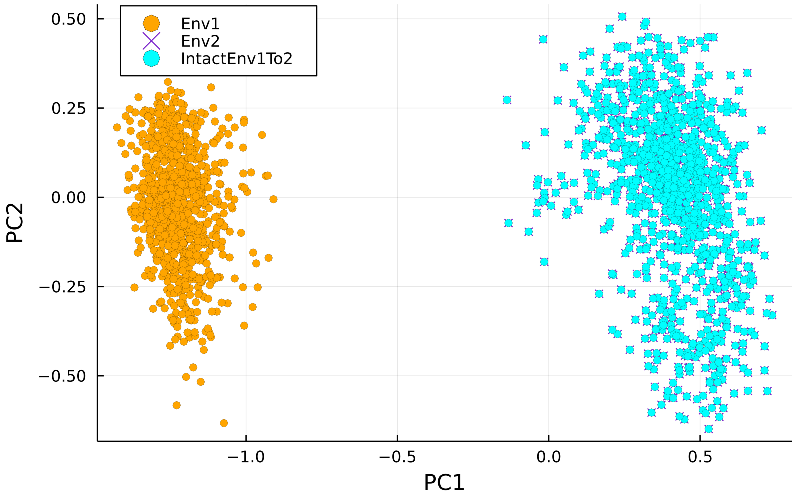

Fig S 4. Phenotypic Pliancy and Fidelity Results from Computational Model without the evolution of PcG Mechanisms: PCA results for all individuals in population at the end of evolution (after generation 1,000) when evolve with no PcG mechanism evolution to assess phenotypic fidelity. After evolution when transfer from environment 1 to environment 2 (cyan) and compare to environment 1 (orange) and environment 2 (purple X's), we see that the individuals move from environment 1 to environment 2 and thus do not exhibit phenotypic fidelity like they do when evolve with polycomb mechanism. Note, when evolve without polycomb and move from environment 1 to environment 2 (cyan), the results exactly overlap with environment 2 (purple X's) because the networks for these cases are the same when do not have any polycomb repressed genes.
