# Supplementary material for: Phenotypic pliancy and the breakdown of epigenetic polycomb mechanisms
Source: PLoS Comput Biol. 2023 Feb 21;19(2):e1010889. doi: 10.1371/journal.pcbi.1010889 (PMC9983867; doi:10.1371/journal.pcbi.1010889)
Supplement: S5 Fig — We measure phenotypic pliancy of a simulated cell by first disrupting the PcG mechanism by removing its control from its repressed genes, we then switch the cell from environment 1 (orange) to environment 2 (purple), we test for stability of the gene expression pattern in this new environment, and finally we determine if this resulting stable gene expression pattern, or phenotype, when break PcG and switch to environment 2 (red) is phenotypically pliant. Mathematically, we first compare if the red moves closer to environment 2 in purple than environment 1 in orange, and then compare if the red is closer to environment 2 in purple than when PcG is intact but switched environments in cyan is to environment 2 in purple. If these conditions are true, then that gene is considered considered phenotypically pliant. Finally, a cell’s phenotypically pliancy score is the percent of genes that move closer to the environment switched to when PcG is broken (red) but remain closer to original environment when PcG is left intact (cyan). (PDF) [file pcbi.1010889.s005.pdf]

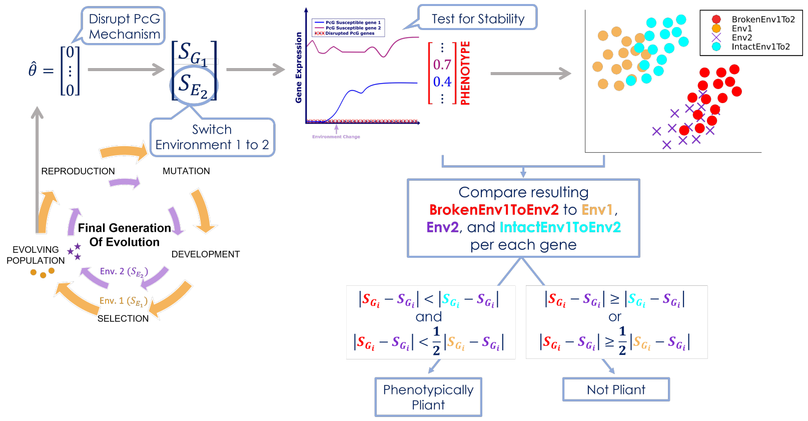

Fig S 5. Schematic for Measuring Phenotypic Pliancy in the Model: We measure phenotypic pliancy of a simulated cell by first disrupting the PcG mechanism by removing its control from its repressed genes, we then switch the cell from environment 1 (orange) to environment 2 (purple), we test for stability of the gene expression pattern in this new environment, and finally we determine if this resulting stable gene expression pattern, or phenotype, when break PcG and switch to environment 2 (red) is phenotypically pliant. Mathematically, we first compare if the red moves closer to environment 2 in purple than environment 1 in orange, and then compare if the red is closer to environment 2 in purple than when PcG is intact but switched environments in cyan is to environment 2 in purple. If these conditions are true, then that gene is considered considered phenotypically pliant. Finally, a cell's phenotypically pliancy score is the percent of genes that move closer to the environment switched to when PcG is broken (red) but remain closer to original environment when PcG is left intact (cyan).
