# Supplementary material for: Phenotypic pliancy and the breakdown of epigenetic polycomb mechanisms
Source: PLoS Comput Biol. 2023 Feb 21;19(2):e1010889. doi: 10.1371/journal.pcbi.1010889 (PMC9983867; doi:10.1371/journal.pcbi.1010889)
Supplement: S6 Fig — The average number of unstable cells in each of the 10,000 different populations when break PRCa alone (blue), PRCb alone (cyan), and PRCa and PRCb together (red). (PDF) [file pcbi.1010889.s006.pdf]

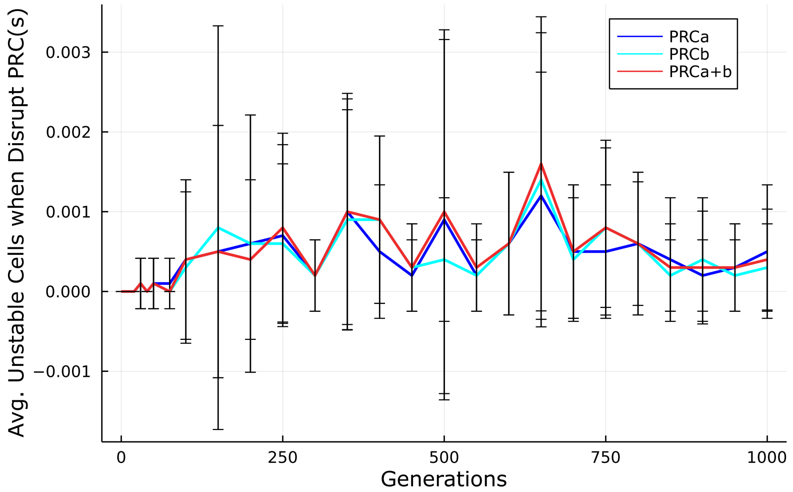

Fig S 6. Average Number of Unstable Cells For Varying Degree of PcG-like Mechanisms Breakage: The average number of unstable cells in each of the 10,000 different populations when break PRCa alone (blue), PRCb alone (cyan), and PRCa and PRCb together (red).
