# Supplementary material for: Phenotypic pliancy and the breakdown of epigenetic polycomb mechanisms
Source: PLoS Comput Biol. 2023 Feb 21;19(2):e1010889. doi: 10.1371/journal.pcbi.1010889 (PMC9983867; doi:10.1371/journal.pcbi.1010889)
Supplement: S7 Fig — Principle Component Analysis (PCA) showing that when PcG-like mechanisms are dysregulated in our model that phenotypic switching is sustainable, such that after switching from environment 1 to environment 2 (red circles) and then back to environment 1 (blue X’s) that the phenotypes more closely resemble what environment they were switched to. As a control, we also see that if break PcG-like mechanisms but keep in environment 1 (green circles) then phenotype stays close to that of the evolved phenotype in environment 1 (orange circles). (PDF) [file pcbi.1010889.s007.pdf]

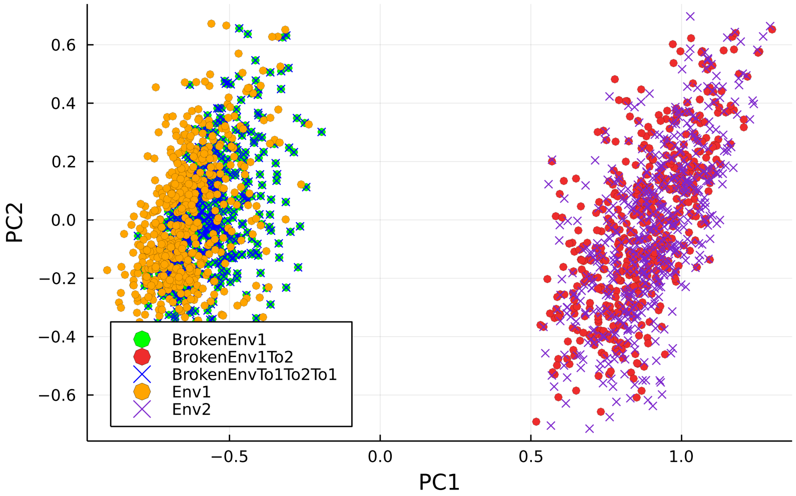

Fig S 7. Sustained Phenotypic Pliancy due to PcG-like Breakdown in Model: Principle Component Analysis (PCA) showing that when PcG-like mechanisms are dysregulated in our model that phenotypic switching is sustainable, such that after switching from environment 1 to environment 2 (red circles) and then back to environment 1 (blue X's) that the phenotypes more closely resemble what environment they were switched to. As a control, we also see that if break PcG-like mechanisms but keep in environment 1 (green circles) then phenotype stays close to that of the evolved phenotype in environment 1 (orange circles).
