# Supplementary material for: Phenotypic pliancy and the breakdown of epigenetic polycomb mechanisms
Source: PLoS Comput Biol. 2023 Feb 21;19(2):e1010889. doi: 10.1371/journal.pcbi.1010889 (PMC9983867; doi:10.1371/journal.pcbi.1010889)
Supplement: S8 Fig — Phenotypic pliancy when PcG-like mechanism is intact versus broken over a wide range of parameters and 10 randomly chosen starting gene-regulatory network architectures. The percent of cells that are phenotypically pliant when PcG-like mechanism is broken is statistically significantly greater than when the PcG-like mechanism remains intact (p-value <0.001). (PDF) [file pcbi.1010889.s008.pdf]

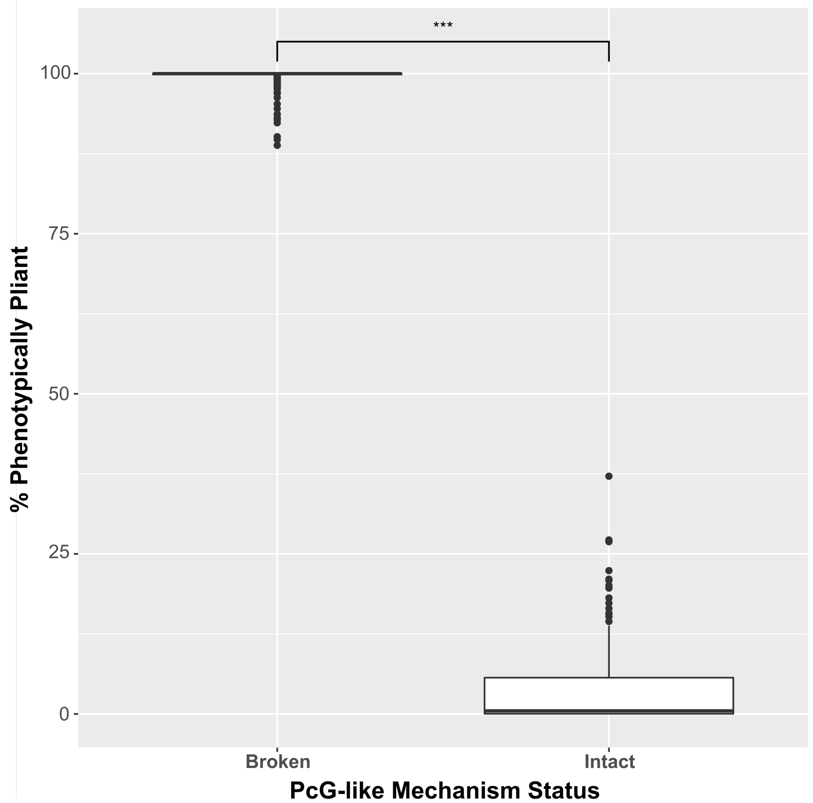

Fig S 8. Overall Phenotypic Pliancy for Model Parameter Testing: Phenotypic pliancy when PcG-like mechanism is intact versus broken over a wide range of parameters and 10 randomly chosen starting gene-regulatory network architectures. The percent of cells that are phenotypically pliant when PcG-like mechanism is broken is statistically significantly greater than when the PcG-like mechanism remains intact (p-value < 0.001).
