# Supplementary material for: Phenotypic pliancy and the breakdown of epigenetic polycomb mechanisms
Source: PLoS Comput Biol. 2023 Feb 21;19(2):e1010889. doi: 10.1371/journal.pcbi.1010889 (PMC9983867; doi:10.1371/journal.pcbi.1010889)
Supplement: S9 Fig — Principle Component Analysis (PCA) results for A. Head and Neck and B. Ovarian metastatic cancer dataset, where metastatic cells’ phenotypes (given by their gene expression patterns) are each represented by dark blue circles, primary cancer cell’s phenotypes are represented by both green and cyan, and the normal non-cancer cells’ phenotypes at the metastatic site and primary site are represented by black and red circles, respectively. Note, the primary cells are split by principle component 2 (y-axis), such that primary cells with PC2 values greater than zero are represented by cyan circles and primary cells with values less than zero are represented by green circles. (PDF) [file pcbi.1010889.s009.pdf]

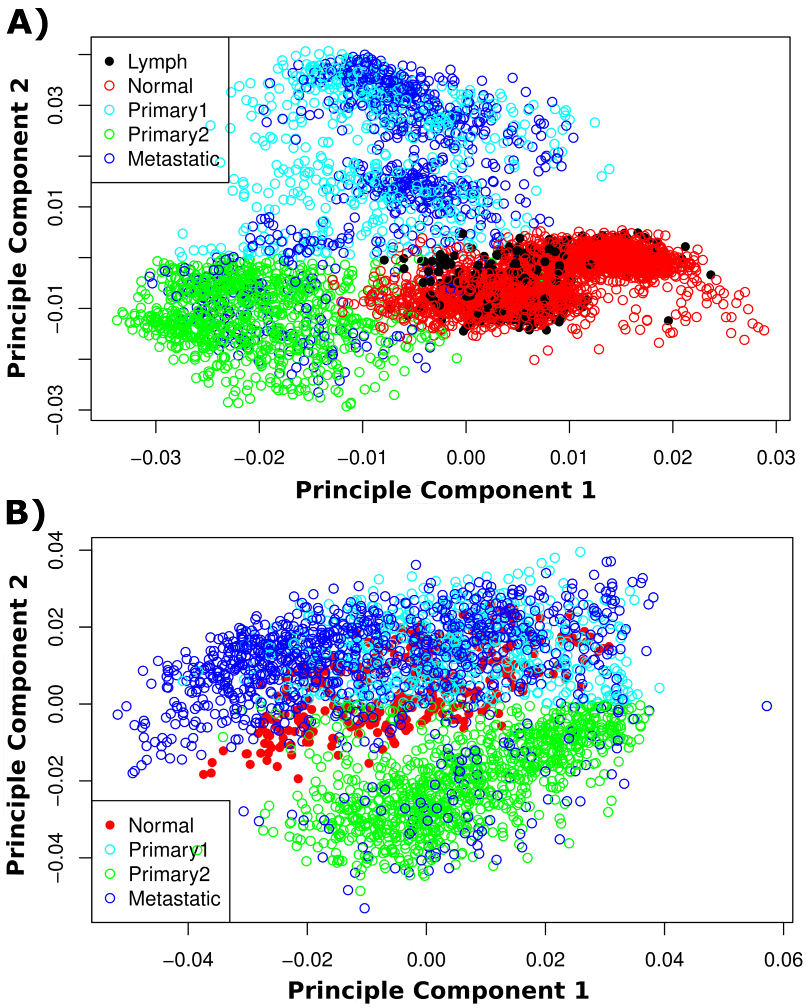

Fig S 9. PCA Results for Metastatic Cancer Datasets: Principle Component Analysis (PCA) results for **A.** Head and Neck and **B.** Ovarian metastatic cancer dataset, where metastatic cells' phenotypes (given by their gene expression patterns) are each represented by dark blue circles, primary cancer cell's phenotypes are represented by both green and cyan, and the normal non-cancer cells' phenotypes at the metastatic site and primary site are represented by black and red circles, respectively. Note, the primary cells are split by principle component 2 (y-axis), such that primary cells with PC2 values greater than zero are represented by cyan circles and primary cells with values less than zero are represented by green circles.
