# Supplementary material for: Phenotypic pliancy and the breakdown of epigenetic polycomb mechanisms
Source: PLoS Comput Biol. 2023 Feb 21;19(2):e1010889. doi: 10.1371/journal.pcbi.1010889 (PMC9983867; doi:10.1371/journal.pcbi.1010889)
Supplement: S10 Fig — Plot of the gene-by-gene differences between primary and normal at the primary site (x-axis) versus the differences between metastatic and normal at the lymph node site or ovary site for the A. head and neck and B. ovarian metastatic cancer dataset, respectively (y-axis). We have included lines y = x and y = −x for comparison. There is a trend for the gene-wise differences between metastatic and normal at the lymph node site being smaller in absolute value than gene-wise differences between primary and normal at the primary site, which is the case for A. 68% of the genes differentially expressed between primary tumor versus metastatic cells (p-value < 10−8) for H&N (shown in red), and B. 72% (p-value < 10−9) for ovarian (shown in red). (PDF) [file pcbi.1010889.s010.pdf]

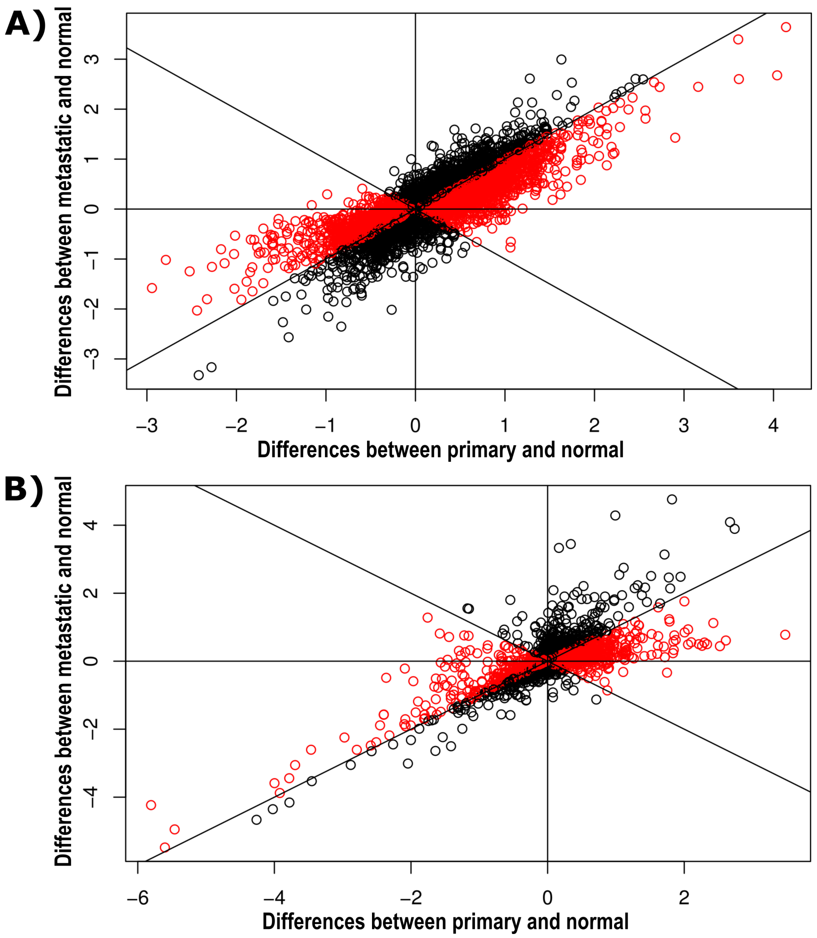

Fig S 10. Gene-Wise Differences for Metastatic Cancer Datasets: Plot of the gene-by-gene differences between primary and normal at the primary site (x-axis) versus the differences between metastatic and normal at the lymph node site or ovary site for the **A.** head and neck and **B.** ovarian metastatic cancer dataset, respectively (y-axis). We have included lines $y = x$ and $y = -x$ for comparison. There is a trend for the gene-wise differences between metastatic and normal at the lymph node site being smaller in absolute value than gene-wise differences between primary and normal at the primary site, which is the case for **A.** 68% of the genes differentially expressed between primary tumor versus metastatic cells (p-value $< 10^{-8}$) for H&N (shown in red), and **B.** 72% (p-value $< 10^{-9}$) for ovarian (shown in red).
