# Supplementary material for: Phenotypic pliancy and the breakdown of epigenetic polycomb mechanisms
Source: PLoS Comput Biol. 2023 Feb 21;19(2):e1010889. doi: 10.1371/journal.pcbi.1010889 (PMC9983867; doi:10.1371/journal.pcbi.1010889)
Supplement: S11 Fig — Histogram of the bootstrapping results, which we perform by randomly picking 1000 samples of PcG mechanism genes and performing linear regression of the score as a function of these genes’ expression profiles for A. H&N and B. ovarian data, respectively. Each plot is the distribution of the positive coefficients computed for each random sample. The red line is the number of PcG mechanism genes with positive coefficients, which is 30 for H&N data and 21 for ovarian data. The mean is 22.3 out of 53 for H&N data, and mean is 10.6 out of 56 for the ovarian data, so the mean for both A. and B. (red line) is statistically significant (p-value < 0.05 and p-value < 10−5, respectively). (PDF) [file pcbi.1010889.s011.pdf]

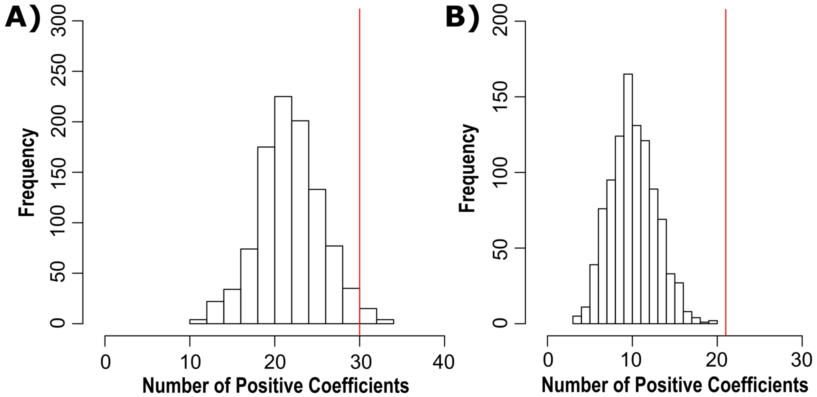

Fig S 11. Significance Assessment of PcG Genes as Source of Pliancy: Histogram of the bootstrapping results, which we perform by randomly picking 1000 samples of PcG mechanism genes and performing linear regression of the score as a function of these genes' expression profiles for **A.** H&N and **B.** ovarian data, respectively. Each plot is the distribution of the positive coefficients computed for each random sample. The red line is the number of PcG mechanism genes with positive coefficients, which is 30 for H&N data and 21 for ovarian data. The mean is 22.3 out of 53 for H&N data, and mean is 10.6 out of 56 for the ovarian data, so the mean for both **A.** and **B.** (red line) is statistically significant (p-value < 0.05 and p-value $< 10^{-5}$, respectively).
