# Supplementary material for: Phenotypic pliancy and the breakdown of epigenetic polycomb mechanisms
Source: PLoS Comput Biol. 2023 Feb 21;19(2):e1010889. doi: 10.1371/journal.pcbi.1010889 (PMC9983867; doi:10.1371/journal.pcbi.1010889)
Supplement: S12 Fig — A. Average phenotypic pliancy score when vary degree of PcG mechanism dysregulation during evolution for all 10,000 populations when move from environment 1 to environment 2 to quantitatively assess pliancy when evolve with stabilizing selection only. We vary degree of dysregulation by breaking PRCa alone (blue), PRCb alone (cyan), or both PRCa and b (red) for all 10,000 populations for a total of 10 million simulated cells pliancy score averaged (y-axis) for different generations throughout evolution (x-axis). B. The average combined fitness for both environment 1 and environment 2 shown in the insert due to scale (green), and the average fitness for environment 1 (red) and environment 2 (blue) fitness results when evolve with stabilizing selection only. (PDF) [file pcbi.1010889.s012.pdf]

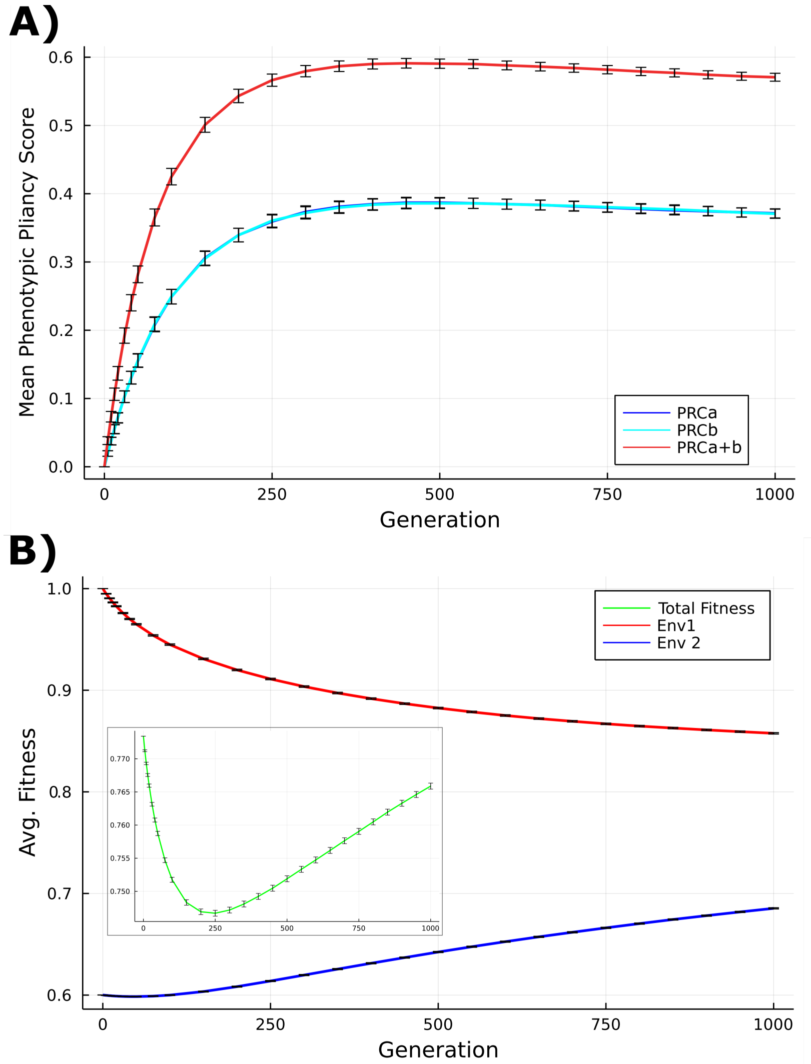

Fig S 12. Phenotypic Pliancy and Fitness Results when Evolve with Stabilizing Selection Only: A. Average phenotypic pliancy score when vary degree of PcG mechanism dysregulation during evolution for all 10,000 populations when move from environment 1 to environment 2 to quantitatively assess pliancy when evolve with stabilizing selection only. We vary degree of dysregulation by breaking PRCa alone (blue), PRCb alone (cyan), or both PRCa and b (red) for all 10,000 populations for a total of 10 million simulated cells pliancy score averaged (y-axis) for different generations throughout evolution (x-axis). **B.** The average combined fitness for both environment 1 and environment 2 shown in the insert due to scale (green), and the average fitness for environment 1 (red) and environment 2 (blue) fitness results when evolve with stabilizing selection only.
