# Supplementary material for: Phenotypic pliancy and the breakdown of epigenetic polycomb mechanisms
Source: PLoS Comput Biol. 2023 Feb 21;19(2):e1010889. doi: 10.1371/journal.pcbi.1010889 (PMC9983867; doi:10.1371/journal.pcbi.1010889)
Supplement: S1 Text — (PDF) [file pcbi.1010889.s013.pdf]

S1 Text

Model Parameter Testing

We test the sensitivity of the model's behavior, and the generality of our results, by measuring the change in the percent of cells exhibiting overall phenotypic pliancy when PcG-like mechanism is intact versus broken over a wide range of parameters and 10 randomly chosen starting gene-regulatory network architectures. Specifically, we vary the following five parameters: gene-regulatory network connectivity density (=0.1, 0.3, 0.5), selection strength (=0.5, 1, 2), gene-activation sigmoidal strength (=1, 4, 6), gene threshold level that triggers PRC repression (=0.1, 0.15, 0.2), and mutation rates (gene mutation rate per genome, environment interaction mutation rate, and PcG-like mechanism mutation rate) (=0.1, 0.2, 0.3). Therefore, we obtain results for 243 different parameter settings. We vary these five parameters independently, and we use the same starting population that undergoes the same 100 different evolutionary trajectories for each parameter setting. We then calculate the percent of phenotypically pliant individuals in each evolved population when PcG-like mechanism is left intact or is broken, then calculate the average for each of these two cases over the 100 different evolved populations for each parameter setting. Given that our phenotypic pliancy score (see Materials and Methods) can only be measured for the individuals with broken PcG-like mechanism, we measure overall phenotypic pliancy for each case when PcG-like mechanism is broken vs. left intact to better compare parameter effects. We measure overall phenotypic pliancy by calculating the distance between its stable phenotype after development in its original environment, S_W, and the resulting stable phenotype upon transferring it post-developmentally to another environment, S'_W. Phenotypic pliancy for a given cell corresponds to a large Euclidean distance $|S_W - S'_W|$, and phenotypic fidelity to a small distance. In our preliminary work, we used a threshold distance of 0.05 to determine phenotypic pliancy, such that if the Euclidean distance is greater than 0.05 then that cell is considered phenotypically pliant. We see a drastic increase in the average phenotypic pliancy when PcG-like mechanism is broken for each of the 243 different parameter settings (p-value = 10^{-16}) (see S8 Fig). Our parameter testing results show the different parameter settings and network architectures do not change our phenotypic pliancy results, strongly suggesting that, while our model lacks biological specificity, it is still biologically relevant in its general implications.

Table A: Polycomb Mechanism Genes Used in Single-Cell RNA-Sequencing Data Analysis:

PcG Protein	TrxG Protein	Controlled by PRC
RING1	CHD8	PCDH15
RNF2	ASH2L	PCDHB1
CBX2	SMARCA1	PCDHB4
CBX4	SMARCA2	PCDHB15
CBX6	RBBP5	CDH8
CBX7	WDR5	CDH13
CBX8	CHD3	CDH18
PCGF1	CHD4	CDH19
PCGF2	CHD1	CDH23
PCGF3	CHD2	RAP1A
BMI1	MTA1	RAP1B
PCGF5	MTA2	RAP1GAP
PCGF6	MTA3	
SCMH1	HDAC1	
RYBP	HDAC2	
YAF2	MBD2	
EZH1	MBD3	
EZH2	POLD3	
EED	BPTF	
SUZ12	SMARCB1	
RBBP4	DPF1	
RBBP7	KMT2A	
	KMT2D	
	KMT2C	
	KMT2B	
	KAT8	
	DPY30	
	SETD1A	
	SETD1B	
	CXXC1	
	WDR82	
	KDM6A	
	NCOA6	
	PAXIP1	
	PAGR1	

Table B: Differential gene expression analysis for metastatic cancer data sets.

Data (Tumor)	DE PcG Genes	Log-Fold Change Sum
**H&N (Primary)**	42 genes	-20.1
**H&N (Metastatic)**	45 genes	-29.2
**Ovarian (Primary)**	25 genes	20.2
**Ovarian (Metastatic)**	16 genes	-14.3

Table C: Model Parameters and Values.

Parameter	Value
Genes	50
Population Size	1,000
Gene Regulatory Network Connectivity	0.1
Generations	1,000
Environment Components	50
Maximum Iterations	100
Gene Mutation Rate	0.1
Environment Interaction Mutation Rate	0.1
PcG-like Mechanism Mutation Rate	0.1
Selection Strength	0.5
Gene-Activation Sigmoidal Strength	1.0
PRC Repression Gene Threshold Level	0.15
PRC Critical Time Point	2
Number Different Environments	2
Number of Different PRCs	2
Proportion of Env. Components Affecting Cell > 0	0.4
Proportion of Genes Able to be Affected by Envs.	0.4
Proportion of Env. Components Affecting Gene	0.4
Minimum Difference Between Env. 1 and Env. 2	70%
Minimum Difference in Env. Optimum States	40%
Individual Weights	Gaussian
Sexual Reproduction Flag	true
